# Cardiac fibrosis following radiation-induced aortitis: an emerging clinicopathologic overlap

**DOI:** 10.1097/MS9.0000000000005062

**Published:** 2026-04-27

**Authors:** Muhammad Ubaid Ur Rehman, Noor Un Nisa, Rafiullah Hotak

**Affiliations:** a Department of Medicine, Gujranwala Medical College, Gujranwala, Pakistan; b Department of Medicine, Jinnah Sindh Medical University, Karachi, Pakistan; c Nangarhar University faculty of Medicine, Jalalabad, Afghanistan

*Dear Editor,*

Cardiac fibrosis in post-radiation aortitis is an arising intricacy of thoracic radiotherapy, representing the overlap of vascular inflammation and myocardial remodeling. This entity is clinically essential because radiation-induced cardiovascular disease affects 10–30% of long-term survivors, often manifesting decades after treatment, with fibrosis serving as a key driver of morbidity^[^1,2^]^. Recognition is essential, as fibrosis is progressive yet potentially modifiable with targeted therapies.

Radiotherapy is typically related to coronary artery disease, valve damage, and pericardial fibrosis. A recent research suggests that radiation-induced aortitis can progress to fibrotic remodeling of the aorta wall, including extension into the myocardium. Endothelial damage, microvascular ischemia, and chronic inflammation contribute to a profibrotic environment, with TGF-β playing a key role. TGF-β activates myofibroblasts and deposits extracellular matrix, whereas CTGF promotes fibrosis downstream. Emerging evidence suggests MicroRNAs like miR-21 further influence this signaling network^[^3–5^]^.

Radiation-induced vasculopathy involving large vessels has been documented, including rare cases of radiation-induced aortitis detected on cross-sectional imaging after chemoradiation for cervical cancer[6]. Myocardial fibrotic remodeling and cardiomyopathy have also been reported as late sequelae of thoracic irradiation, confirmed with cardiac magnetic resonance and histopathology[7]. Longitudinal imaging studies show progressive diffuse myocardial fibrosis following radiotherapy for breast cancer[8].

Radiation-related fibrosis is rarely seen alone; it is usually coupled with valvular disease, restrictive cardiomyopathy, coronary atherosclerosis, and pulmonary fibrosis^[^3,4^]^. Thyroid disease is very common in thoracic irradiation survivors, exacerbating late morbidity.

Therapeutic strategies are still in the experimental stage. Preclinical efficacy has been shown for the anti-TGF-β drugs fresolimumab and pamrevlumab, whereas nintedanib, a tyrosine kinase inhibitor that is approved for pulmonary fibrosis, may have cardiac uses[9]. Although the long-term effects are not always constant, pentoxifylline and vitamin E have demonstrated benefits in reducing radiation fibrosis[10]. Lifelong surveillance with cardiac magnetic resonance imaging, echocardiography, and biomarkers (TGF-β, CTGF, and miR-21) is advised for early diagnosis due to the decades-long latency. This article aligns with the TITAN Guidelines on the need for transparency in AI use in healthcare[11].

Cardiac fibrosis developing after radiation-induced aortitis should be recognized as a separate clinical condition that connects vascular injury with myocardial damage. Detecting it early, using biomarker-based surveillance, and testing antifibrotic treatments may help improve long-term outcomes in cancer survivors. Current evidence is largely observational and extrapolated, highlighting the need for systematic characterization.

## Data Availability

Not applicable.
